# A New Method of Pivoting Teeth

**Published:** 1877-10

**Authors:** 


*A New Method of Pivoting Teeth.*—Dr. Bonwill, in the March meeting of the New York Odontological Society, read a paper on Pivot Teeth, in which he introduces and advocates a method apparently advantageous in at least some cases, and possesses decided merit in that it is simple and comparatively inexpensive. The process is simply to enlarge somewhat the opening in the root, having previously treated if necessary, making the canal in the root of such a shape as will retain firmly a filling. An ordinary plain plate tooth is now fitted to this root, with a gold backing upon the pins and a cap of gold to fit the prepared end of the root, these are soldered together, and a hole drilled in the part of the plate directly opposite the root canal, which hole is countersunk on its outer side. A platinum pin is prepared of a size which will pass easily through the hole drilled in the plate. The pin should be long enough to reach from the bottom of the cavity in the root, to a little beyond the outer surface of the plate which is intended to fit over it; this pin has a thread cut on it the entire length, and the lower end is flattened somewhat, to prevent its turning. It is now bent at an obtuse angle backward, that there may be more room for a nut to turn. The prepared pin is placed in position in the cavity, amalgam is packed around it, and allowed to set, care being taken that the relation of the pin to the hole in the tooth is preserved. The operation is concluded by placing a little gutta-percha on the surface of the backing or cap which comes in contact with the stump, and screwing a conical nut down upon the threaded screw, the nut being so prepared as to fit neatly into the countersunk plate. The nut should have a crucial (+) slot in it, that it may be more easily screwed home, and will require tightening after a few days, when it is permanently finished, and may be covered over, if any inequalities occur, with os artificiel. The pin should be platinum, not gold, as the latter would be rendered brittle by the mercury in the amalgam. [We suggest the alloy of platinum and iridium as stronger.—[*Ed. American Journal of Dental Science.*]

A modification of this process is adapted to molars and bicuspids.

